# Genetic Variations in Two Seahorse Species (*Hippocampus mohnikei* and *Hippocampus trimaculatus*): Evidence for Middle Pleistocene Population Expansion

**DOI:** 10.1371/journal.pone.0105494

**Published:** 2014-08-21

**Authors:** Yanhong Zhang, Nancy Kim Pham, Huixian Zhang, Junda Lin, Qiang Lin

**Affiliations:** 1 Key Laboratory of Tropical Marine Bio-resources and Ecology, South China Sea Institute of Oceanology, Chinese Academy of Sciences, Guangzhou, China; 2 Vero Beach Marine Laboratory, Florida Institute of Technology, Vero Beach, Florida, United States of America; VIB & Katholieke Universiteit Leuven, Belgium

## Abstract

Population genetic of seahorses is confidently influenced by their species-specific ecological requirements and life-history traits. In the present study, partial sequences of mitochondrial cytochrome b (*cytb*) and control region (CR) were obtained from 50 *Hippocampus mohnikei* and 92 *H. trimaculatus* from four zoogeographical zones. A total of 780 base pairs of *cytb* gene were sequenced to characterize mitochondrial DNA (mtDNA) diversity. The mtDNA marker revealed high haplotype diversity, low nucleotide diversity, and a lack of population structure across both populations of *H. mohnikei* and *H. trimaculatus*. A neighbour-joining (NJ) tree of *cytb* gene sequences showed that *H. mohnikei* haplotypes formed one cluster. A maximum likelihood (ML) tree of *cytb* gene sequences showed that *H. trimaculatus* belonged to one lineage. The star-like pattern median-joining network of *cytb* and CR markers indicated a previous demographic expansion of *H. mohnikei* and *H. trimaculatus*. The *cytb* and CR data sets exhibited a unimodal mismatch distribution, which may have resulted from population expansion. Mismatch analysis suggested that the expansion was initiated about 276,000 years ago for *H. mohnikei* and about 230,000 years ago for *H. trimaculatus* during the middle Pleistocene period. This study indicates a possible signature of genetic variation and population expansion in two seahorses under complex marine environments.

## Introduction

Examining patterns of genetic diversity, population structure, and expansion has become an important part in the management plans of endangered populations, and population size is the major determinant of population well-being and extinction risk [1]. In marine environments, population genetics are often impacted by species-specific ecological requirements and life-history traits [2]. The complex and dynamic interactions between the physical and biological environment and the physiology, behaviour, and life histories of individual taxa can apparently lead to the differentiation of marine populations [3].

For marine species, climatic events can undoubtedly impact their historical biogeography; however, marine patterns are relatively poorly known because of the high geological complexity and biological diversity [4]. Based on the endemism of the marine biota in the Northwest Pacific Ocean, three zoogeographical zones have been identified, i.e., the Oriental Zone, Japan Warm-Temperate Zone, and the Tropical Zone [5]. These three zones are defined largely by ecological rather than by historical factors [5]. Sea surface temperature has been postulated to be the primary factor, which governs the formation of these zoogeographical zones, rather than other environmental factors [5].

In marine environments, some species that have long-lived, free-swimming, and feeding (planktotrophic) larval phases probably have relatively high dispersal abilities, and this promotes genetic exchange between populations [2]. However, seahorses are at the lower end of the marine fish dispersal continuum and retain historical patterns [6]. All seahorses are vulnerable to habitat damage because of their feeble swimming ability and small home range behaviour [7]. Many seahorse species undergo a planktonic newborn stage between two and six weeks, after which they settle down into sessile habitats [7], [8]. A long planktonic period is likely to create widespread gene flow across geographically disconnected populations, resulting in genetic homogeneity [9].

The three-spot seahorse *Hippocampus trimaculatus* and Japanese seahorse *H. mohnikei* are the most abundant and economically important seahorse species along China's coast. *H. trimaculatus* has a wide distribution range throughout the tropical and sub-tropical regions in Southeast Asia and is about 8–15 cm in body length [10], [11]. *H. mohnikei* is a small body size (about 5–8 cm in body length) and inshore-water species, and is generally found in seagrass areas less than 10 m deep [12], [13]. The distribution of *H. mohnikei* is limited in the Northeast Asian Sea [13]. It is important to know the population structure in order to conserve these and other seahorse species because of heavy exploitation and environmental changes. Here, we demonstrated the mtDNA diversity among *H. trimaculatus* and *H. mohnikei* along China's coast by sequence analyses of cytochrome *b* (*cytb*) and control region (CR) haplotypes. We then compared the characteristic modes of their genetic variations and the evidence for population expansion across past climatic events.

## Materials and Methods

### Sample collection

**Figure 1:**
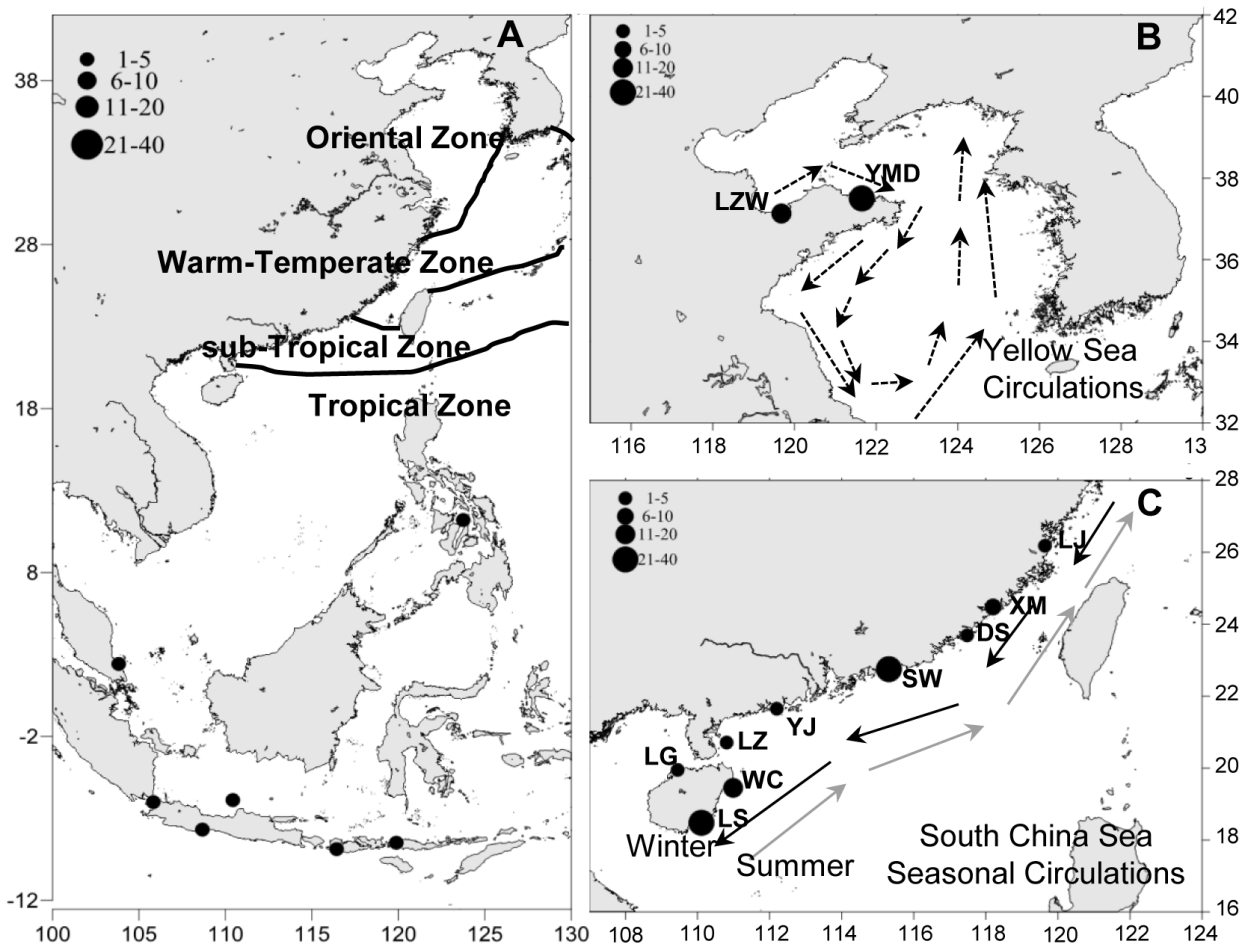
Sampling localities and sizes of *H. mohnikei* and *H. trimaculatus*. The zoogeographical zones are modified by the previous report of Briggs [7] (names in the figure A, and zones are delineated by black lines). Alphabetical letters indicate the seahorse groups: (A) outgroup for *H. trimaculatus*; (B) *H. mohnikei* group (under the influence of the Yellow Sea circulations (dotted arrows) [48]); and (C) *H. trimaculatus* group (under the influence of overall seasonal circulation in the South China Sea [black arrows in winter and grey arrows in summer] [44], [45]).

A total of 50 *H. mohnikei* were sampled from Yangmadao and Laizhouwan along North China's coast, which belonged to the Oriental Zone (OZ) [3], and a total of 92 *H. trimaculatus* individuals were sampled from nine localities along Southeast China's coast, and all samples were pooled into three groups from three zoogeographical zones, i.e., Warm-Temperate Zone (WTZ), sub-Tropical Zone (sTZ), and Tropical Zone (TZ) [3] (Table 1 and Fig. 1). Most specimens of *H. mohnikei* and *H. trimaculatus* were collected by researchers on board trawl boats, and a few were obtained with the help of local fishermen and buyers; all seahorses were immediately preserved until DNA isolation. Seahorse project is a key study in the South China Sea Institute of Oceanology, and also a key research focus in the Chinese Academy of Sciences. Seahorses used in this experiment have been absolutely approved for the use of research work, and sampling areas in our study are public, and there is no special policy to protect the seahorses. Some research work in our laboratory aims to obtain detailed information about wild seahorses and then provide data that may lead to the protection of seahorses in some areas. All seahorse samples utilized in this study have received animal ethics approval for experimentation by the Chinese Academy of Sciences. We have provided a scanned certificate for our investigation on seahorses.

**Table 1:** Sampling location and sample size (n) of *H. mohnikei* and *H. trimaculatus*.

	Location	Latitude	Longitude	n
*H. mohnikei*				
LZW	Laizhouwan	37.13°N	119.68°E	20
YMD	Yangmadao	37.50°N	121.65°E	30
*H. trimaculatus*				
Warm-Temperature Zone (WTZ)	—	—	—	15
LJ	Lianjiang	26.18°N	119.64°E	5
XM	Xiamen	24.48°N	118.20°E	6
DS	Dongshan	23.69°N	117.48°E	4
sub-Tropical Zone (sTZ)	—	—	—	35
SW	Shanwei	22.75°N	115.31°E	33
YJ	Yangjiang	21.65°N	112.20°E	1
LZ	Leizhou	20.71°N	110.81°E	1
Tropical Zone (TZ)	—	—	—	42
LG	Lingao	19.95°N	109.45°E	2
WC	Wenchang	19.45°N	110.99°E	15
LS	Lingshui	18.48°N	110.11°E	25

### Molecular analysis

A small amount of tissue from the tail of each seahorse was removed and macerated using phosphate buffered saline (PBS) buffer for extraction. The macerating tissue and muscle from the fresh seahorses were frozen in liquid nitrogen and then ground into powder. Genomic DNA was extracted using the AxyPrep Multisource Genomic DNA Miniprep Kit (Axygen Biosciences, USA) following the manufacturer's protocol with minor modifications: the tissue homogenate was incubated at 56°C for 2 hours during cell lysis with Proteinase K. All DNA samples were stored at -80°C until polymerase chain reaction (PCR) amplification. A part of the mitochondrial *cytb* gene (895 base pairs) was amplified employing the seahorse specific primers: forward shf 5′-CTACCTGCACCATCAAATATTTC-3′ and reverse shr2 5′-CGGAAGGTGAGTCCTCGTTG-3′ [6]. DNA amplification of CR sequences followed the methodology published previously [27]. All PCR reactions were carried out in a total volume of 50 µl, utilizing 3 µl (10-100 ng) DNA, 0.25 µl Taq DNA polymerase (5 U/µl, TaKaRa, China-Japan Joint Company, Dalian, China), 1 µl of each primer (10 µM), 4 µl dNTP Mixture (2.5 mM), 5 µl Ex Tag Buffer (10×), and 35.75 µl ddH_2_O. The thermocycling sequence was conducted as follows: an initial step of 94°C (3 min); a second step of 35 cycles of 94°C (30 s), 50°C (30 s), and 72°C (75 s); and a final step of 72°C (10 min). The primers and amplification conditions used for CR were as described in Teske et al. [27]. Amplified PCR products were checked on 1.5% agarose gels and purified for sequencing using the E. Z. N. A. Gel Extraction Kit (Omega, USA). *Cytb* genes and CR were commercially sequenced using PCR purified products from both forward and reverse primers (BGI, China). Sequences were assembled and edited using Bioedit 7.0.9.0 [14], and subsequently aligned utilizing ClustalW [15]. Sequences were submitted to GenBank (accession numbers: *cytb* for *H. trimaculatus* KC519325-KC519363 and *H. mohnikei* KC527556-KC527584; CR for *H. trimaculatus* KJ158359-KJ158392 and *H. mohnikei* K J158393-KJ158419).

### Population genetic analyses

Genetic diversity indices (based on composition and transition/transversion bias) were calculated using MEGA5 [16]. The numbers of individuals (*n*), number of variable sites (*ns*), number of haplotypes (*np*), haplotype diversity (*h*), nucleotide diversity (π), and average number of nucleotide differences (*k*) for each species' population were estimated using the software DnaSP 5.10.00 [17].

### Demographic reconstruction

Pairwise mismatch distributions, sum of square deviations (SSD), and raggedness index (R) were performed using Arlequin 3.1 [18] for all sampling locations combined to find evidence of past demographic expansion. According to coalescent theory, a population at demographic equilibrium usually exhibits a multimodal mismatch distribution, but is usually unimodal following a recent population demographic or range expansion [19]. If the test statistics show no significant SSD value and low R value, it means that the population has experienced sudden expansion [20]. We also tested the neutral theory in Arlequin 3.1 employing Tajima's *D* [21] and Fu's *Fs* [22]. Tajima's test is the most conservative test of neutrality; whereas, Fu's *Fs* is the most powerful test for population growth. Expectations of Fu's *Fs* and Tajima's *D* are significantly negative values (P<0.05) in a sudden expansion population [22]. The relationship Tau  = 2ukt/g was used to estimate the time of expansion (t), where k is the number of nucleotides assayed; u is the mutation rate per nucleotide; and g is the generation interval. An average mutation rate of 6.3×10^−9^ per site per year for the seahorse *cytb* gene was assumed based on a generation time of approximately 1 year [23]. To analyze population structure, an analysis of molecular variance (_AMOVA_) was utilized in the Arlequin 3.1 software. We also used the Bayesian skyline plot (BSP) [24] implemented in _BEAST_ [25] to assess historical changes in effective population size. An uncorrelated lognormal relaxed clock was employed for *cytb* alignment. Divergence time was estimated to be 2% per million years (My) based on the entire mtDNA molecule, which was widely used for bony fish [26], [27].

### Phylogenetic analyses

The *cytb* sequences were subjected to the phylogenetic analysis based on a neighbor-joining (NJ) tree and the maximum likelihood (ML) method in PAUP 4.0b10 [28]. We selected the best-fit nucleotide substitution model for each locus using the Akaike information criterion (AIC) in the program Modeltest 3.0 [29]. *H. kuda* (JX217831.1) was used as an outgroup for *H. mohnikei. H. trimaculatus* sequences (AY322461, AY322457, AY322452, AY322437, AY322435, AY322454, AY322462, AY322471, AY322473, AY322467, AY322475, AY322468, AY322464, and AY322434) were obtained from GenBank as additional reference sequences for phylogeography analysis. Polymorphic sites were determined using DnaSP 5.10.00, and these sites were utilized to construct an unrooted median-joining haplotype network using software package Network 4.6.1.0 [30].

## Results

### DNA sequence variability and genetic diversity

A total of 780 bp of the *cytb* fragment were unambiguously sequenced in 50 specimens of *H. mohnikei* and 92 specimens of *H. trimaculatus*. The mean number of nucleotide composition in *H. mohnikei* was T = 27.1%, C = 15.5%, A = 34.0%, and G = 23.4%; in *H. trimaculatus*, it was T = 28.1%, C = 15.4%, A = 30.4%, and G = 26.1%. The transition and transversion rates were 1.77 and 8.82, respectively. Of the 780 bp nucleotide sequence of *H. mohnikei*, there were 25 variable sites, accounting for 3.2% of the length of the sequence. Of these variable sites, there were 12 parsimony informative sites, accounting for 48.0% of the length of the sequence. In *H. trimaculatus*, the number of variable sites was 45, accounting for 5.7% of the length of the sequence, and the number of parsimony informative sites was 10, accounting for 22.2% of the variable sites.

Of the 50 *H. mohnikei cytb* sequenced, there were 29 unique haplotypes (Hj1-Hj29), which were distributed across the two sampled populations as follows: 22 haplotypes from Yangmadao and 11 from Laizhouwan. There were four haplotypes shared by the two populations. The overall haplotype (*h*) and nucleotide diversity (π) in *H. mohnikei* was 0.946 and 0.00353, respectively (Table 2). Of the 92 *H. trimaculatus cytb* sequenced, there were 39 unique haplotypes (Ht1-Ht39), which distributed across the three sampled population pools as follows: seven haplotypes from WTZ, 17 from sTZ, and 24 haplotypes from TZ. The overall haplotype (*h*) and nucleotide diversity (π) in *H. trimaculatus* was 0.902 and 0.00281, respectively (Table 2). Of the *H. mohnikei* and *H. trimaculatus* CR sequenced, there were 27 haplotypes (JC1-JC27) and 34 haplotypes (TC1-TC34), respectively.

**Table 2:** Genetic diversity of mitochondrial cytochrome *b* for *H. mohnikei* and *H. trimaculatus*.

		n	ns	np	h	π	k
*H. mohnikei*	YMD	30	19	22	0.961±0.023	0.00340±0.00036	2.65517
	LZW	20	16	11	0.912±0.056	0.00368±0.00080	2.87500
	All	50	25	29	0.946±0.023	0.00353±0.00039	2.75763
*H.trimaculatus*	WTZ	15	7	7	0.872±0.067	0.00217±0.00038	1.69231
	sTZ	35	21	17	0.899±0.034	0.00273±0.00037	2.12773
	TZ	42	29	24	0.919±0.028	0.00307±0.00035	2.39489
	All	92	45	39	0.902±0.020	0.00281±0.00023	2.19401

Numbers of individuals (*n*), number of segregating sites (*ns*), number of haplotypes (*np*), haplotype diversity (*h*), nucleotide diversity (π), and average number of nucleotide differences (*k*).

### Phylogenetic analyses

**Figure 2:**
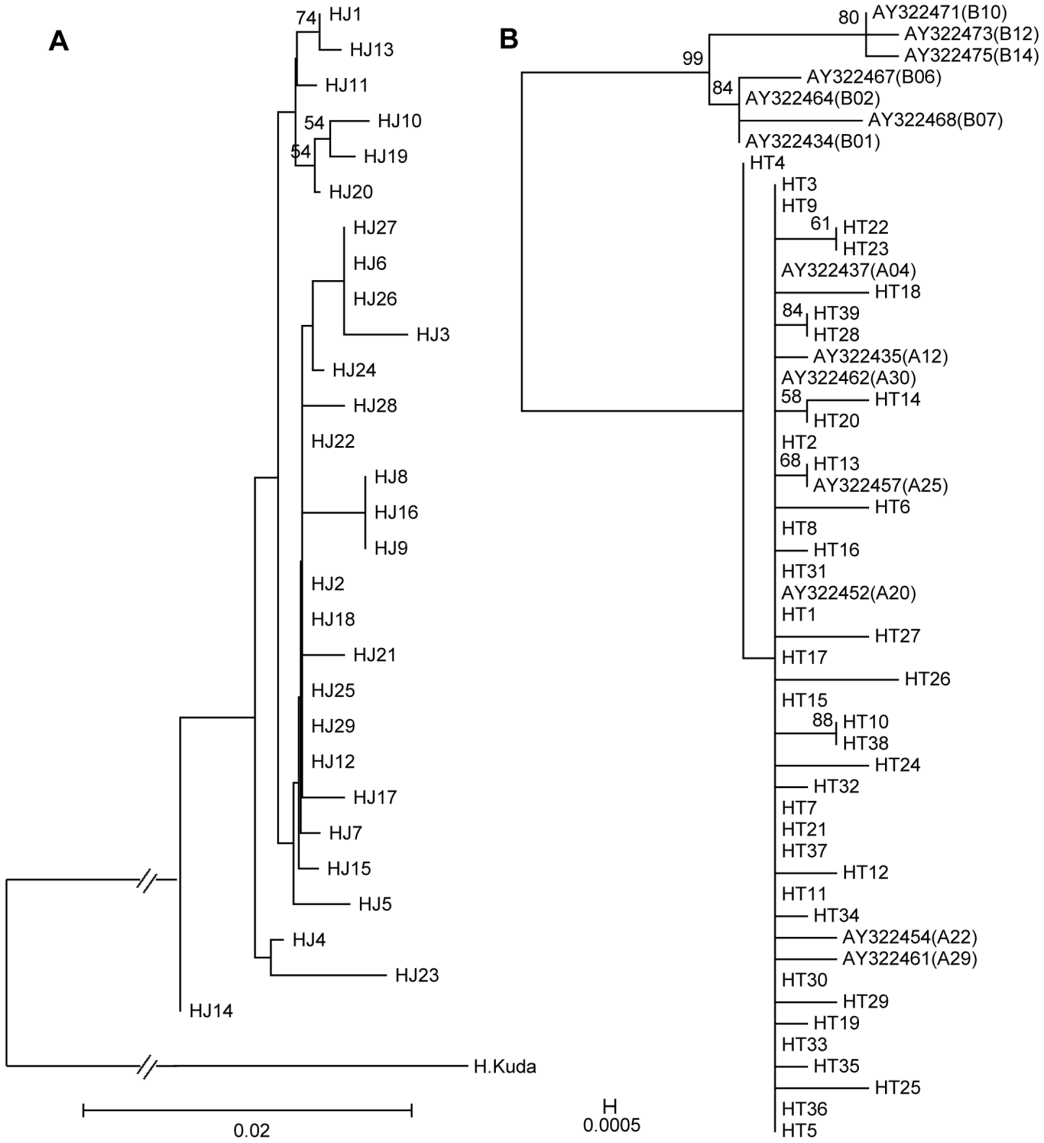
Phylogenetic relationships among *H. mohnikei* and *H. trimaculatus* based on *cytb* genes. NJ tree generated from *H.mohnikei* haplotypes, plus one outgroup (*H. kuda*) (A) and ML tree generated from *H. trimaculatus* haplotypes (B). Cytochrome b sequences are compared to GenBank sequences from reference samples. Figures on branches indicate the degree of bootstrap support (only values above 50% bootstrap support are illustrated).

The relationships of haplotypes based on *cytb* was determined using a neighbor-joining (NJ) algorithm for *H. mohnikei* (Fig. 2A) and maximum likelihood (ML) method for *H. trimaculatus* (Fig. 2B) with bootstrap values indicated above each branch. Partitions with <50% support were not shown. Sequences representing the major clusters of mtDNA haplotypes detected in a recent survey of a diverse sample of lineage A and lineage B (west and east of Wallace's Line, respectively) for the three-spot seahorses from Southeast Asia [6] are included in Fig. 2 and labeled according to their GenBank accession numbers. The haplotypes A4, A12, A22, and A30 (from Malaysia), A20, A25, and A29 (from Java) were almost clustered together with all of the haplotypes in this study. However, haplotypes B1, B2, B6, B7, B10, B12, and B14 were clustered into another branch, which was unlike A lineage. All of the *H. trimaculatus* haplotypes grouped with the A lineage, suggesting an origin on the Asiatic side of Wallace's Line.

**Figure 3:**
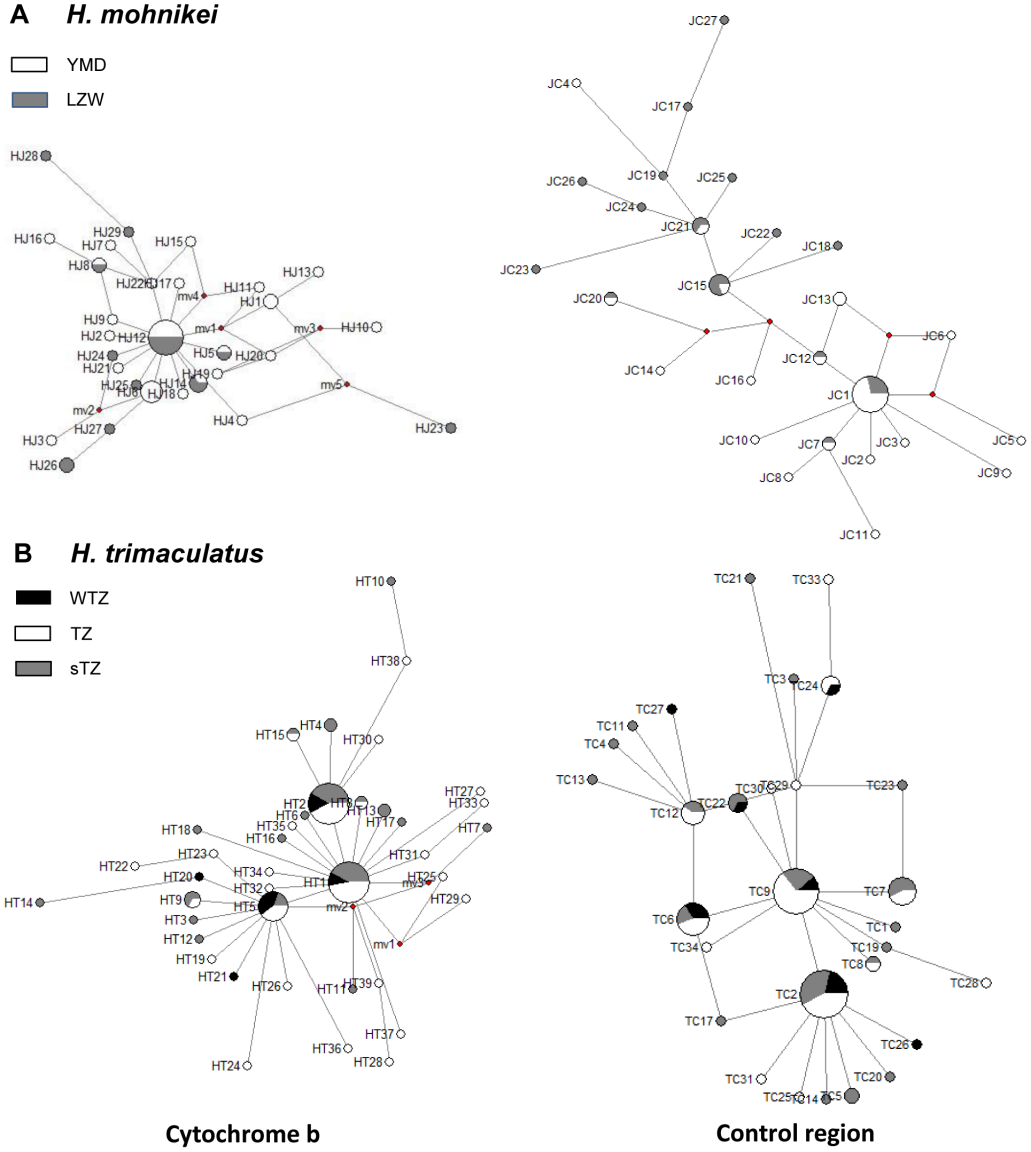
Statistical parsimony network showing phylogenetic relationships among *H. mohnikei* (A) and *H. trimaculatus* (B) haplotypes. The area of each circle is proportional to the number of specimens sharing that haplotype. Small open squares represent hypothesized intermediate haplotypes not observed in our sample.

The median-joining network among haplotypes of *H. mohnikei* populations presented a star-like distribution trend. For the *cytb* haplotype network, the highest frequency haplotype was Hj12, occupying a central position in the network (Fig. 3A). Other haplotypes were associated with Hj12 by spur. Based on the coalescent theory, the *H. mohnikei* populations experienced a significant population expansion [31]. Because haplotype Hj12 is in the basal position of the network, it was the most widely distributed haplotype and may be the ancestor haplotype. For the CR haplotype network, the highest frequency haplotype was JC1. The *H. mohnikei* haplotype networks were consistent with the NJ phylogenetic tree. For *H. trimaculatus*, the network suggested little or no association between haplotypes geographically, which was consistent with the ML phylogenetic tree. For *cytb* haplotype network, the highest frequency haplotype was Ht1 and Ht2, followed by Ht5, which consisted of 19, 19, and 10 individuals, respectively, and they occupied a central position in the network (Fig. 3B). For the CR haplotype network, the highest frequency haplotype was TC2 and TC9, which both consisted of 17 individuals (Fig. 3B).

### Population structure

AMOVA analyses identified that there was not a strong geographic subdivision between the two populations of *H. mohnikei* sampled. Only 1.85% of the total variance was attributed to differences among populations. However, 98.15% of the total variance was attributed to differences within populations. Fixation index further supports a rise in gene flow with *Φ_ST_*  = 0.01849 (P>0.05) indicating no genetic structure. AMOVA analyses also showed that there was not a strong geographic subdivision between the three populations of *H. trimaculatus* sampled (*Φ_ST_* = 0.00032, P>0.05).

### Historical demography

**Figure 4:**
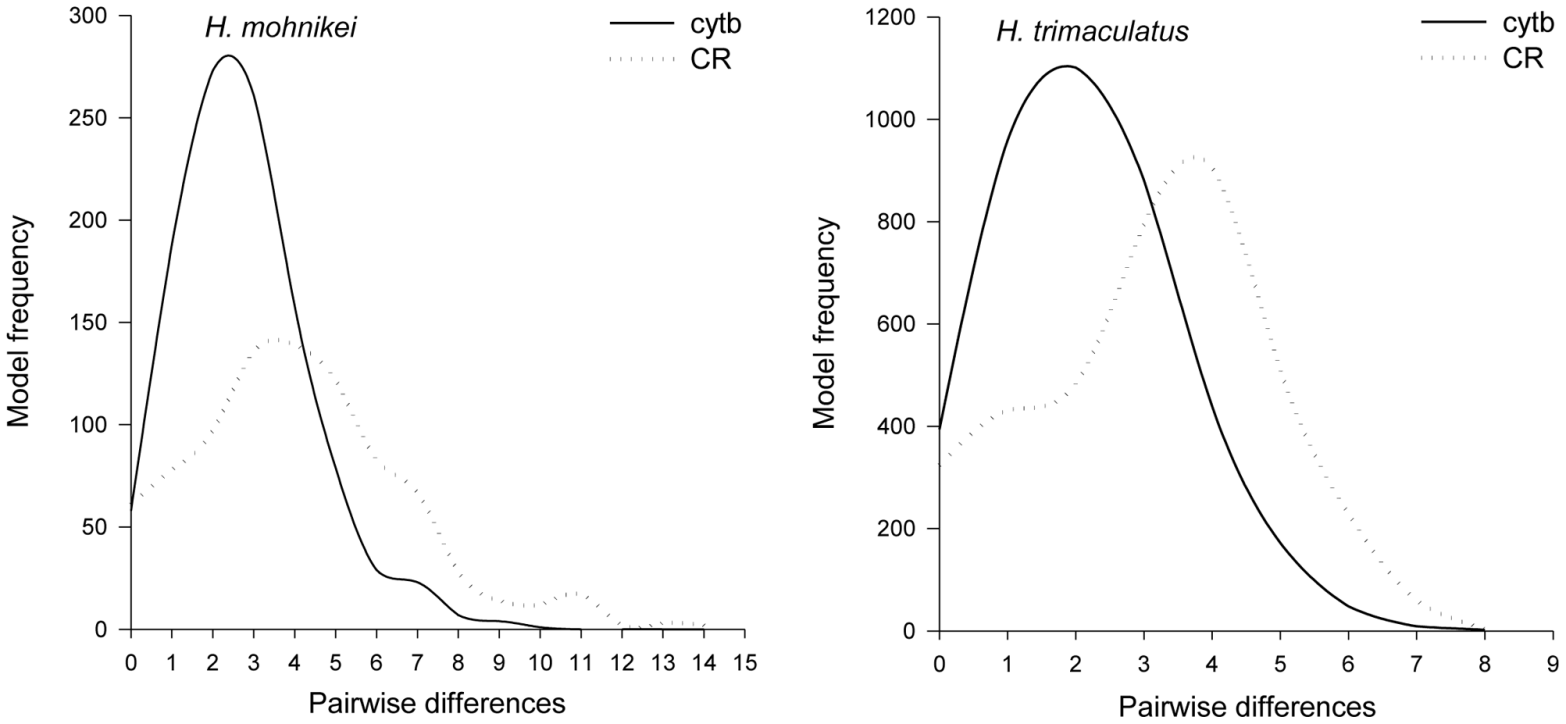
Mismatch distributions for *cytb* and CR sequences found in *H. mohnikei* and *H. trimaculatus*. In each case, the curve represents the observed frequency of pairwise differences among haplotypes.

**Figure 5:**
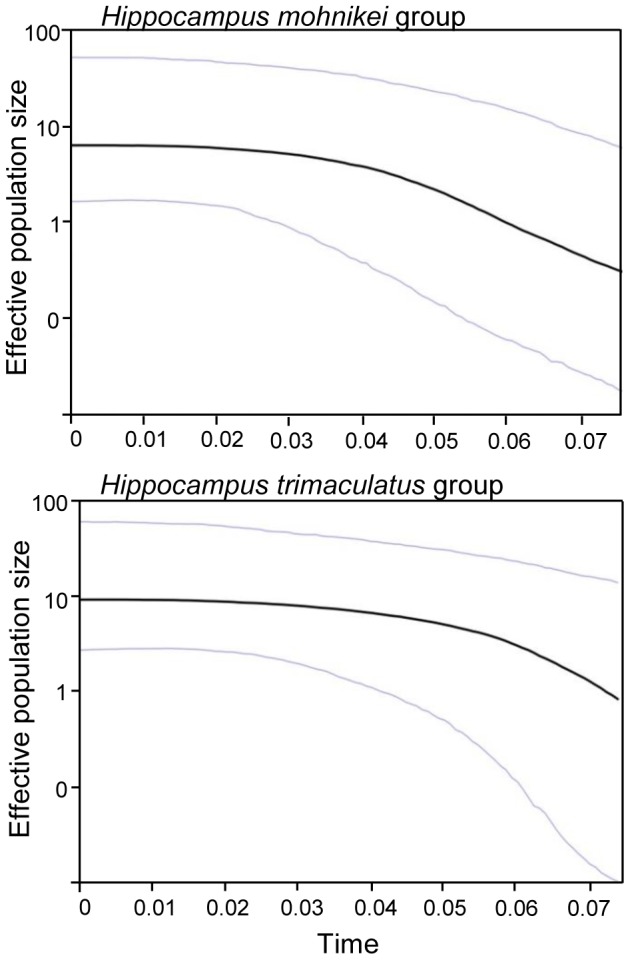
Bayesian skyline plots representing historical demographic trends of *H. mohnikei* and *H. trimaculatus* in China. The mean estimate is enclosed within the 95% highest posterior densities.

Demographic history changes were analyzed for *H. mohnikei* and *H. trimaculatus* populations using neutrality tests and mismatch distributions. Tajima's *D* and Fu's *Fs* values in *H. mohnikei* were significantly negative in all populations. For *H. trimaculatus*, Tajima's *D* and Fu's *Fs* values were significantly negative (P<0.05) in all populations, except that Tajima's *D* was not significant in the WTZ group (Table 3). Due to significantly negative Tajima's *D* and Fu's *Fs* values for all seahorse populations combined in the present study (except for negative, but not significant Tajima's *D* values for the WTZ population), we speculate that the populations may have experienced population expansion in the past. The *cytb* and CR data sets exhibited a unimodal mismatch distribution (Fig. 4), which indicated that both seahorse species may have undergone a recent population demographic or range expansion. At the same time, a small and not statistically significant SSD and R-value showed population expansion (Table 3). Estimates of the time since the start of population expansion of the *H. mohnikei* ranged from about 276,000 years ago, while *H. trimaculatus* populations in China started expanding approximate 230,000 years ago; the oldest dates were for TZ, and the youngest were for WTZ (Table 3). The results of BSP (Fig. 5) also rejected population stability for both species. BSP estimates of *H. mohnikei* group suggested that the population has expanded about 11-fold, from about 0.55 to about 6. BSP estimates of *H. trimaculatus* group suggested that the population has expanded about 7-fold, from about 1 to about 7.

**Table 3:** Demographic statistics for mitochondrial cytochrome *b* of *H. mohnikei* and *H. trimaculatus*.

		Tajima'sD		Fu's Fs		Mismatch Distribution		
		D	P	D	P	SSD	Raggedness index	Tau
*H. mohnikei*	YMD	−1.54678	0.04500	−21.79502	0.00000	0.00786	0.05820	2.76562
	LZW	−1.52178	0.04500	−5.04335	0.00300	0.00322	0.02595	2.78125
	TOTAL	−1.69266	0.02500	−26.46206	0.00000	0.00093	0.03759	2.71484
*H.trimaculatus*	WTZ	−0.94729	0.18800	−2.83361	0.02200	0.00631	0.08087	1.85156
	sTZ	−1.98345	0.00800	−11.83494	0.00000	0.00074	0.04849	2.12500
	TZ	−2.19551	0.00200	−22.30991	0.00000	0.00062	0.03645	2.46875
	TOTAL	−2.40148	0.00000	−27.17594	0.00000	0.00064	0.04186	2.25586

Neutrality tests: Tajima's *D*, Fu's *Fs*, and expansion (coalescence) time under the sudden expansion assumption in mutation-generations (τ); D: values of Tajima' *D* and Fu's *Fs*; P: p-values of Tajima' *D* and Fu's *Fs*; SSD: sum of the square deviations.

## Discussion

### Genetic diversity and structure

In the present study, the haplotype diversity at a high level and the nucleotide diversity in the lower-middle-level indicated that *H. mohnikei* and *H. trimaculatus* populations may have experienced a long period of stable evolution, or there were different lineages along China's coast. This pattern of high haplotype diversity is common in marine fish and consistent with previous studies of *Sardina pilchardus* [32], *Schizothorax prenanti* [33], *H. trimaculatus* [6], [34] *H. ingens* [35], and *Hoplostethus atlanticus* [36]. High haplotype diversity at a gene locus within populations may have also been caused by other factors, such as large population size, environmental heterogeneity, life-history traits, origin, as well as ages of the species [37]. The pattern of genetic variability with high haplotype diversity, but relatively low nucleotide diversity, suggests that the population has undergone population expansion [38]. Genetic variability is considered to be the foundation of evolution and can be affected by many factors, such as mutation rates, effective population size, and gene flow [39]. Gene flow is a constraint on local genetic differentiation, and the adaptation between populations and low gene flow between populations can lead to genetic subdivision of populations [40], [41].

An advantage of using *cytb* over nuclear genes is that an mtDNA gene tree can yield insights into population history that may be lost due to recombination in a nuclear gene tree. That an NJ tree separated the *cytb* haplotypes with high bootstrap support indicates distinct genetic structuring between the east and west coast populations of seahorses along India's coasts [34]. As shown in Fig. 2, due to the low levels of genetic variation present between our sampled populations, the NJ tree generated from *H. mohnikei* haplotypes and ML tree generated from *H. trimaculatus* haplotypes had low support values, implicating no obvious genetic structure in *H. mohnikei* and *H. trimaculatus* populations. Many bootstrap values for nodes were low, indicating that the substructure within the major clusters is uncertain.

AMOVA analyses indicated the absence of significant population genetic differentiation across *H. mohnikei* and *H. trimaculatus* populations. *H. trimaculatus* has the most widespread distribution range, indicating potentially high dispersal capabilities; whereas, *H. mohnikei* is confined to China's Bohai Sea and Yellow Sea, indicating potentially low dispersal capabilities.

Population structure is affected by genetic drift, local adaptation, and gene flow. In a marine environment, the development of population structure is confidently influenced by factors that affect dispersal, such as ocean currents, historical variance, and geographic distance coupled with differences in dispersal ability and habitat discontinuity [35]. The possible explanation for the homogeneity of populations of *H. mohnikei* and *H. trimaculatus* was the high level of gene flow. Although the mobility of seahorses is feeble, marine currents make passive dispersal possible. Overall seasonal circulation in the South China Sea is cyclonic in winter and anticyclonic in summer, with a few stable eddies [42], [43]. The seasonal circulation is mostly driven by monsoon winds, and also related to water exchange between the South China Sea and the East China Sea through the Taiwan Strait, and between the South China Sea and the Kuroshio Current through the Luzon Strait (Fig. 1) [42], [43]. Several other fish species in the area, which have pelagic larval and/or juvenile stages, show genetic homogeneity among populations and could be passively transported by ocean currents [44], [45].

The Yellow Sea circulations play an important role in the passive dispersal of *H. mohnikei*. The eastward Lubei coastal current flows along the northern part of the Shandong Peninsula, and then turns south in Chengshanjiao; however, northeastward currents in the Lunan coast flows from southwest to northeast all year round (Fig. 1). At the same time, there is an offshore mesoscale anticyclonic in Qingdao-Shidao [46]. Therefore, the coastal currents of the Yellow Sea might have limited the dispersal range of *H. mohnikei*. On the other hand, even if there are a few number of *H. mohnikei* migrating to the South China Sea with the ocean currents, the environment may not be suitable for inhabitation of the population, such as the water temperature. These ecological differences often result in varying dispersal, which plays an important role in determining the phylogeographical structure of marine species. The Sea of Japan, East China Sea, and South China Sea have been isolated during the glacial periods [47]. Recent molecular studies indicated that some widespread marine species exhibited phylogeographical patterns corresponding to these three glacial refugia [48]. Geographic boundaries during the Pleistocene may also have played an important role in these species dispersion.

### Population expansion

The population of *H. mohnikei* displayed a genetic pattern typical of a population that has undergone a recent population expansion due to its one common haplotype present across the range, most haplotypes unique to single sites, and a pattern of a shallow star-shaped haplotype network. As shown in Fig. 3B, the distribution of the central and abundant haplotype Ht1 and Ht2, extended from WTZ to TZ, which supports that *H. trimaculatus* in this region has undergone range expansion. The range expansion was a recent phenomenon and may not have obtained the migration-drift equilibrium, as shown by the lack of phylogeographical structure [38]. A similar star-like pattern of genetic relatedness among haplotypes was seen in other seahorses, such as *H. hippocampus* (L. 1758) [49].

Due to significantly negative Tajima's *D* and Fu's *Fs* values for all seahorse populations combined in the present study (except for negative, but not significant Tajima's *D* values for the WTZ population), we speculate that the populations may have undergone population expansion in the past. Furthermore, mismatch distributions were calculated for *H. mohnikei* and *H. trimaculatus* to investigate the hypothesis of a population expansion. Previous studies have revealed that population bottlenecks and population expansions have a significant effect on the pattern of genetic polymorphism among haplotypes in the population [19]. These theoretical studies demonstrate that populations in stable demographic equilibrium have a multimodal mismatch distribution (ragged and chaotic); whereas, the distribution appears unimodal after recent demographic expansions [19], [50]. The mismatch distributions for *H. mohnikei* and *H. trimaculatus* populations were unimodal and fully consistent with a population expansion. The expansion was initiated 276,000 years ago for *H. mohnikei* and 230,000 years ago for *H. trimaculatus* during the middle Pleistocene period. The Pleistocene, which spans from about 1.6 Myr to 10,000 years before the present, was punctuated by a series of large glacial-interglacial changes [51]. It was probably a result of a high dispersal potential, which was particularly advantageous during the rising of sea water temperatures and levels [52], [53], [54]. Responding to the climatic events, marine ecosystems make corresponding changes in species distributions, and abundances and productivity [55]. Glaciation-interglaciation events and associated changes in the marine environment probably have had great effects in the demographic history of many marine and coastal fish, such as *Beryx decadactylus* [45], *Hoplostethus atlanticus* [36], and *Glyptocephalus stelleri* [56].

## Conclusions

This study demonstrates the genetic variation and population expansion for two seahorses, *H. mohnikei* and *H. trimaculatus*, which are small feeble swimming fish and confidently influenced by their species-specific ecological requirements and life-history traits. Both seahorses have experienced population expansions since the mid-Pleistocene, and the population span of the expansion of *H. mohnikei* is larger and occurred earlier than that of *H. trimaculatus*. The observed lack of population differentiation can be explained by this past population expansion and present-day juvenile or sub-adult dispersal. Our study detected the absence of significant genetic divergence across the South China Sea in *H. trimaculatus*, suggesting that broad-scale conservation management strategies may be appropriate for this species. As a connective study on seahorses, future work will aim to assess the stability of the genetic variation and population expansion in the near future with the possible impact from heavy exploitation of seahorses and environmental change along China's coast.
